# Disease transmission through expiratory aerosols on an urban bus

**DOI:** 10.1063/5.0037452

**Published:** 2021-01-12

**Authors:** Zhihang Zhang, Taehoon Han, Kwang Hee Yoo, Jesse Capecelatro, André L. Boehman, Kevin Maki

**Affiliations:** 1 Department of Naval Architecture and Marine Engineering, University of Michigan, 138 NAME Bldg, 2600 Draper Drive, Ann Arbor, Michigan 48109-2145, USA; 2 Department of Mechanical Engineering, University of Michigan, 2045 AL (W.E. Lay Auto Lab), 1231 Beal, Ann Arbor, Michigan 48109-2121, USA; 3 Department of Mechanical Engineering, University of Michigan, 2011 AL (W.E. Lay Auto Lab), 1231 Beal, Ann Arbor, Michigan 48109-2121, USA; 4 Department of Naval Architecture and Marine Engineering, University of Michigan, 210 NAME Bldg, 2600 Draper Drive, Ann Arbor, Michigan 48109-2145, USA

## Abstract

Airborne respiratory diseases such as COVID-19 pose significant challenges to public transportation. Several recent outbreaks of SARS-CoV-2 indicate the high risk of transmission among passengers on public buses if special precautions are not taken. This study presents a combined experimental and numerical analysis to identify transmission mechanisms on an urban bus and assess strategies to reduce risk. The effects of the ventilation and air-conditioning systems, opening windows and doors, and wearing masks are analyzed. Specific attention is paid to the transport of submicron- and micron-sized particles relevant to typical respiratory droplets. High-resolution instrumentation was used to measure size distribution and aerosol response time on a campus bus of the University of Michigan under these different conditions. Computational fluid dynamics was employed to measure the airflow within the bus and evaluate risk. A risk metric was adopted based on the number of particles exposed to susceptible passengers. The flow that carries these aerosols is predominantly controlled by the ventilation system, which acts to uniformly distribute the aerosol concentration throughout the bus while simultaneously diluting it with fresh air. The opening of doors and windows was found to reduce the concentration by approximately one half, albeit its benefit does not uniformly impact all passengers on the bus due to the recirculation of airflow caused by entrainment through windows. Finally, it was found that well fitted surgical masks, when worn by both infected and susceptible passengers, can nearly eliminate the transmission of the disease.

## I. INTRODUCTION

The COVID-19 pandemic has affected people throughout the world, and recovery from the pandemic depends upon a detailed understanding of how transmission occurs through the various ways humans interact in society. It is known that among the different pathways of transmission, a dominant mode is that airborne particles carry the virus from person to person.^1^ To date, transmission of SARS-CoV-2 has predominantly taken place in indoor spaces, especially those with poor ventilation.^2,3^ It is therefore not surprising that the COVID-19 pandemic poses significant challenges to public transportation. The primary focus of this study is on the factors that contribute to disease transmission on urban buses.

The most documented case of COVID-19 transmission on a bus is from an outbreak on a long-distance coach on January 22 in Hunan, China.^4^ Security cameras showed that the contagious individual had not interacted with others on the bus, yet 8 of the 45 passengers were infected over the 4-h journey. Moreover, a passenger was infected who boarded 30 min after the contagious passenger disembarked. A similar situation occurred in Zhejiang province around the same time as the Hunan event.^5^ 128 individuals traveled on two buses to a worship event in Eastern China. It was determined that those who rode the bus with air recirculation enabled had an increased risk of infection compared with those who rode a different bus. It was suggested that airborne transmission may partially explain the increased risk of SARS-CoV-2 infection among the passengers.

Urban buses are an important part of many public transportation systems and are unique from coach buses in that trips are typically much shorter (tens of minutes), the passengers may be standing or seated, and they make frequent stops. Although urban buses are heavily used in urban and suburban areas throughout the world, the transmission of airborne particles on urban buses has received little attention.

The shedding of virus-laden particles is a complicated biological process by which mucus lining the lungs contains the virus, and as air passes through the respiratory tract, small droplets are formed and pass through the mouth and into the surrounding air. The droplets vary in size, from sub-micron to greater than 50 *µ*m.^6^ The virus shedding rate is a fundamental quantity that defines the rate at which the virus becomes airborne, yet it is difficult to quantify. The process depends on the individual’s breathing rate, which varies from person to person, and for an individual depends on the activity level, such as resting, walking, speaking, singing, shouting, coughing, and sneezing.^7^ The analysis of a superspreading event at a choral rehearsal in the state of Washington in the USA estimates the rate to be around 970 quanta/h.^8^ In addition, key factors such as the location of the virus within the respiratory tract and the quantity of virus influence the contagiousness of airborne droplets. While it is difficult to directly measure, recent studies^9^ indicate that the shedding rate *λ* is in the range of 1 < *λ* < 50 s^−1^.

To date, the vast majority of simulation efforts to predict exposure to droplet transmission consider computational fluid dynamics (CFD) where the turbulent air flow is solved using Reynolds-averaged Navier–Stokes (RANS) coupled with Lagrangian particle tracking. Recent examples include transmission of pathogen-laden expiratory droplets (with specific attention to the novel coronavirus; SARS-CoV-2) on buses,^10–13^ office buildings,^14^ in hospitals,^15^ and outdoor environments.^16^ CFD of aerosol transmission in buses have been studied to assess the influence of filtration ventilation modes, relative humidity (RH), seat arrangement, among other factors, in the context of SARS-CoV-2,^12^ influenza,^10^ and air pollutants.^11,13^ Yang *et al.*^12^ performed numerical simulations to assess the impact of ventilation modes and relative humidity on droplet transmissions in a coach bus. They considered 14 passengers and droplets of two sizes (10 *µ*m and 50 *µ*m) with five air conditioning supply directions. It was found that ventilation, relative humidity (RH), and initial droplet size significantly influence transmission. It was recommended that high RH, backward supply direction, and seating passengers at nonadjacent seats can effectively reduce the risk of infection through droplet transmission in buses. Another CFD study analyzed the effects of window openings on self-pollution for a school bus.^11^ It was found that opening the driver’s window could increase exhaust through the window and door gaps in the back of the school bus, while opening windows in the middle of the bus could mitigate this phenomenon. Increasing the driving speed was also found to promote higher ventilation rates and further dilute the air. While these studies provide important insight into factors contributing to transmission rates on buses, detailed analyses are limited, especially on urban buses. Even more, experimental measurements of aerosol transmission on buses remain elusive.

The purpose of this study is to investigate the transport of aerosols through an urban bus to identify key factors that contribute to disease transmission and provide guidelines for mitigation strategies. Experiments are performed to investigate the transport of polydisperse droplets under different settings of the air conditioning system. The experiments also quantify the influence of opening the doors or the windows of the bus. High resolution CFD simulations are performed to determine the transport of small (<5 *µ*m) particles and investigate the role of the air-conditioning, the location of the infected passenger, the role of face coverings, and the effects of opening the windows and doors. A risk metric is defined based on the number of particles exposed to susceptible passengers.

## II. DESCRIPTION OF THE URBAN BUS, RISK OF RIDING, AND MITIGATION STRATEGIES

**FIG. 1.**
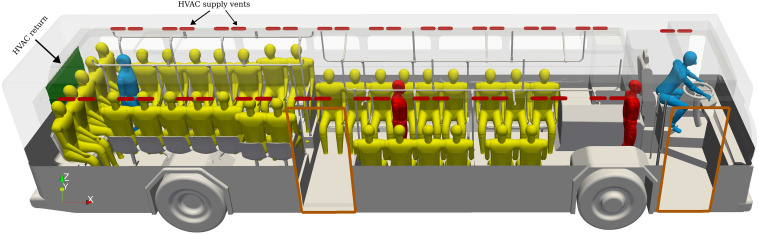
Perspective view of the urban bus interior.

In this work, an urban bus that is used on the campus of the University of Michigan is studied. The capacity of the bus is 35 passenger seats, with room for up to additional 30 standing passengers. The bus makes frequent stops of ∼30 s–60 s, every one to four minutes. The longest ride from terminus to terminus of any one of the newly redesigned bus routes is 15 min.^17^ The bus dimensions are 12.1 × 2.58 × 2.95 m^3^ (*L* × *W* × *H*) and a rendering of the bus is shown in Fig. 1.

The airflow within the bus is affected by the air-conditioning system (heating, ventilation, and air conditioning—HVAC), the opening of windows and doors, breathing, thermal effects, and passenger movement when loading and unloading. The HVAC system can provide a maximum flow rate of 2500 ft^3^/min (70.8 m^3^/min), and the interior volume of the bus is ∼2000 ft^3^ (56.6 m^3^). The HVAC flow rate and bus interior volume correspond to ∼60 air-changes/h. The single ventilation fan draws air from within the passenger compartment through a return vent and adds 20% fresh air from outside before returning the air to the passenger compartment through supply vents. The HVAC return and supply vents are shown in Fig. 1. The orientation of the HVAC supply vents is such that air exits vertically downward. The dimension of each supply vent is 9 in by 1 in (0.229 × 0.0254 m^2^) and that of the single return vent is 4 ft × 1.5 ft (1.22 × 0.457 m^2^). A total of 42 supply vents are located along both sides of the bus ceiling, a pair of which are directly above the driver seat.

There are 14 windows that open, including one near the driver. The opening part of each window is 10 inches by 3 feet and 8 inches, or 0.25 meters by 1.09 meters. There are forward and rear loading doors on the passenger side of the bus. A transparent shield door is installed between the driver and passenger areas to impede virus transmission between the two areas so that only the rear door is used for loading and unloading.

Much of the work toward mitigation in public spaces is based on the distance that should be kept between people, commonly referred to as social distancing. The early work^18^ demonstrates that the larger heavier particles, those greater than 100 *µ*m, fall within 2 m of being exhaled. This principle is used throughout the world for socially distancing guidelines, but it does not account for the influence of convection of the small particles that travel with the ambient air currents. Furthermore, it is becoming clear that very small particles, those that do not fall to the ground, stay suspended in air and travel passively with the ambient air flow.^1^ In order to safely use urban buses, it is important to understand virus transport via the smallest particles so that effective mitigation strategies can be implemented. Toward this goal, in this paper, high resolution numerical simulations are conducted to predict the travel of particles that are subjected to all the relevant forces that govern its transport through the passenger cabin, with particular attention to the turbulent flow that dominates the transport of the small aerosols.

The risk associated with riding a bus is quantified by directly calculating the number of inhaled particles at each location on the bus with contagious passengers located in different positions. Additionally, the role of masks is demonstrated by using a simple model of mask effectiveness based on the recent literature.^19^ Finally, the influence of using the variable speed HVAC system and opening the windows and doors is quantified.

The infected passenger is characterized as shedding the virus at the highest suggested rate^9^ of 50 s^−1^. This number is based on the literature and analysis of several spreading events in Asia and Europe. The shedding rate represents a worst-case scenario corresponding to a highly contagious passenger speaking loudly and continuously throughout the bus ride. It is assumed that only one infected passenger is present, and the analysis investigates transmission with the infected passenger either standing in the front or in the middle of the bus.

## III. EXPERIMENTAL ANALYSIS

### A. Experimental setup

One aerosol generator and two sampling instruments were utilized to measure aerosol transport and dispersion and emulate an infected passenger on an urban bus. A theatrical fog machine (CO-Z Portable Fog Machine, 400 W) was used to generate aerosols using a water-based “fog juice.” The nontoxic water-based fog juice is comprised of deionized water, propylene glycol [C_3_H_8_O_2_, complete active space (CAS) number 57-55-6], and triethylene glycol (C_6_H_14_O_4_, CAS number 112-27-6). The injection time of all cases was 3 s to generate sufficient mass and consistent concentration of the aerosol.

**FIG. 2.**
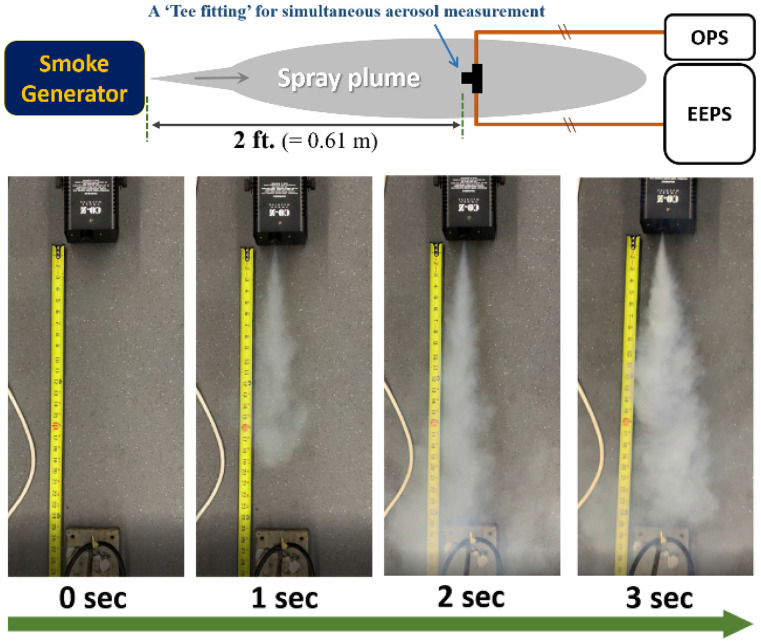
Schematic and pictures of sampling configuration for the evaluation of size distribution from the smoke generator.

The target range of the aerosol size measurement was from 5 nm to 10 *µ*m (10 000 nm) to include the size of the virus itself, virus-containing aerosols, and droplets. Two different types of instruments were used in this study: (i) a TSI EEPS (Engine Exhaust Particle Sizer) Model 3090 for measuring nano-sized aerosol size and numbers and (ii) a TSI OPS (Optical Particle Sizer) Model 3330 for quantifying the micro-sized aerosol size and numbers. The two instruments were connected at a unified sampling location via a tee fitting, as shown in the upper schematic diagram in Fig. 2.

The EEPS instrument measures the number and size distribution of aerosols from the 6 nm to 520 nm range with a high temporal resolution (10 Hz), which permits instant visualization of aerosol dynamics during transient events. The TSI Optical Particle Sizer (OPS) Model 3330 is a portable instrument that also provides a fast measurement of aerosol concentration and size distribution using an optical particle counting technology. The OPS instrument has a size range from 0.3 *µ*m to 10 *µ*m with the 1 Hz time resolution.

The two instruments were mounted in a stacked configuration on a cart to permit movement to different sampling locations on the bus. Thus, the release of aerosol in different locations of the bus was enabled by the use of the portable smoke generator, and the sampling at different locations in the bus enabled assessment of aerosol transport times and aerosol dilution throughout the bus. The instruments were benchmarked each time using ambient aerosols and a high-efficiency particulate air (HEPA) filter (99.97% capture for particles larger than 3 *µ*m) for accurate measurements.

Both the aerosol measuring instruments count the particle numbers in a specific range of the aerosol diameter. The EEPS measures 22 electrometer channels and draws 32 aerosol diameter sizes from 6 nm to 520 nm, while the OPS measures 16 ranges of aerosol diameters from 0.3 *µ*m to 10 *µ*m. A conversion equation is necessary to calculate the total concentration for each instrument for each bin that comprises a particle size range. The calculation method for total concentration (total number) used for the EEPS and OPS data processing is given by

$$N=\int_{D_{p1}}^{D_{p2}}\frac{dN}{d\log D_{p}}d\log D_{p},$$ (1)

where *D*_*p*_ is the channel midpoint of the particle diameter and *N* represents the concentration in a specific range of diameter (*D*_*p*_). *D*_*p*1_ and *D*_*p*2_ are the target range of the aerosol total concentration. In this study, the EEPS used the *D*_*p*1_ and *D*_*p*2_ values as 6 nm and 520 nm, and the OPS used 300 nm to 10 *µ*m for containing the maximum range of the instrumental diameter size windows. The total concentration is expressed as a concentration size spectral density in *dN*/*d* log *D*_*p*_ (cm^−3^) with units of *N* (cm^−3^). The logarithmic term arises from the fact that the size classes are logarithmically spaced. In order to convert *dN*/*d* log *D*_*p*_ (cm^−3^) to *N* (cm^−3^), the *dN*/*d* log *D*_*p*_ values of interest were summed and divided by the number of channels for each instrumental value.

**FIG. 3.**
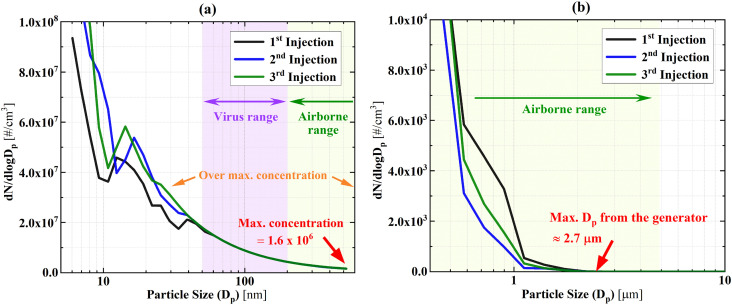
Smoke generator emitting aerosol size distribution and concentrations: (a) EEPS—nanorange and (b) OPS—microrange.

Prior to conducting the experiments, the aerosols emitted from the smoke generator were measured using the two instruments. Figure 2 illustrates a schematic of aerosol generation and the measurement setup (top figure) and images of the smoke plume at different instances in time (bottom pictures). Figure 3 shows the results of the aerosol size distribution and concentrations from the smoke generator spray plume (including three repeated measurements). Figure 3(a) is the nanorange aerosol EEPS result, and Fig. 3(b) is the microrange aerosol OPS result. The particle concentration over 30 nm diameter shows consistent results for all three experiments, and it is due to the exceeded maximum concentration limit of the EEPS instrument. The OPS results in Fig. 3(b) also showed a maximum concentration as the particle size approaches 500 nm diameter, and the smoke generator emitted a maximum particle diameter around 2.7 *µ*m, as indicated by the arrow. Based on the literature, the virus size is around 50 nm–200 nm^20,21^ [purple shadowed area in Fig. 3(a)], and the virus carrying aerosol size is up to 5 *µ*m [green shadowed area in Figs. 3(a) and 3(b)]. Thus, the aerosols are sufficient for representing the target aerosols in this study.

### B. Experimental results

**FIG. 4.**
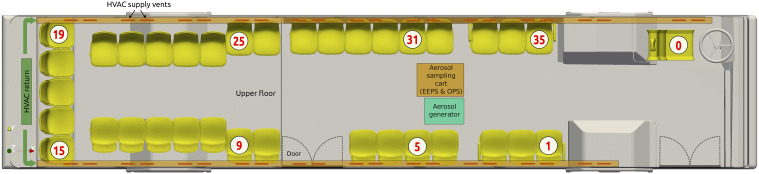
Schematic of the experiment setup.

Measurements were taken to assess the influence of the location on the bus and the effects of having the windows open or closed. Each condition is classified by the locations of aerosol sampling and injection. The sample location is denoted as A, B, or C, corresponding to the driver (seat 0), front passenger (seat 31), or middle passenger (seat 9), as depicted in Fig. 4. The three injection locations are denoted 1, 2, or 3, corresponding to the front (seat 5), middle (seat 9), or back (seat 15). Table I summarizes the experimental measurement locations.

**TABLE I.** Experimental measurement locations.

No.	Case	Sampling seat	Injection seat
1	Ambient	No. 0 (driver)	None
2	A–1	No. 0 (driver)	No. 5 (front)
3	A–2	No. 0 (driver)	No. 9 (middle)
4	A–3	No. 0 (driver)	No. 15 (back)
5	B–1	No. 31 (front)	No. 5 (front)
6	B–2	No. 31 (front)	No. 9 (middle)
7	B–3	No. 31 (front)	No. 15 (back)
8	C–1	No. 9 (middle)	No. 5 (front)
9	C–2	No. 9 (middle)	No. 9 (middle)
10	C–3	No. 9 (middle)	No. 15 (back)

Table II shows the detailed geometry of sampling and injection points, and the measured distance (*D*) and height (*h*) values are from the sidewall and the floor on the bus. The direction of aerosol injection and sampling followed the passenger and driver face direction of the seats, so the front (1, B) and middle (2, C) seats are toward the bus central direction and the driver (A) and back (3) seats are toward the front direction, as shown in Fig. 4.

**TABLE II.** Sampling and injection location (*D*—distance from the sidewall and *h*—height from the floor).

Type	Location	Seat No.	Distance (D)	Height (h)
Sampling (A)	Driver	No. 0	21.0 in (0.53 m)	35.0 in (0.89 m)
Sampling (B)	Passenger–front	No. 31	31.0 in (0.79 m)	42.0 in (1.07 m)
Sampling (C)	Passenger–middle	No. 9	31.0 in (0.79 m)	45.0 in (1.14 m)
Injection (1)	Passenger–front	No. 5	22.0 in (0.56 m)	20.5 in (0.52 m)
Injection (2)	Passenger–middle	No. 9	25.0 in (0.64 m)	21.5 in (0.55 m)
Injection (3)	Passenger–back	No. 15	23.5 in (0.60 m)	23.5 in (0.60 m)

The baseline experiments were conducted in a stationary bus with the windows and doors closed. The aerosol numbers and concentration are sensitive to the ambient environment (such as temperature, pressure, and humidity), so each case of experiments was repeated at least two times for all conditions and on different days for the baseline experiments. In this study, the aerosol response time was calculated as the time difference between the aerosol injection and the initial slope change in the total concentration in all cases.

**FIG. 5.**
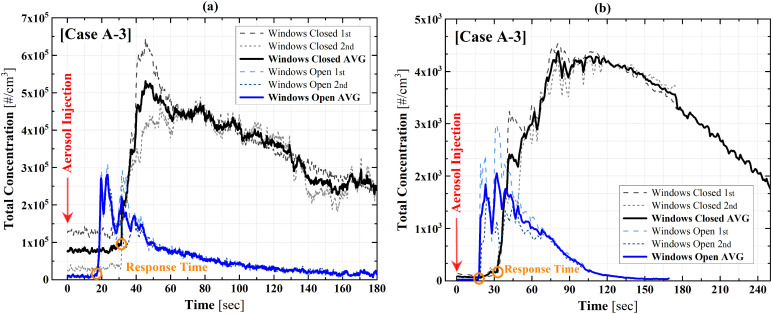
Total concentrations with and without windows open: (a) nanosized aerosols and (b) microsized aerosols. Case A-3.

Figure 5 shows the time history of the aerosol concentration measured with both instruments for case A-3 when the bus is stopped, but the windows are either open or closed. The measurements are repeated two times, and the average is shown in the dark line. The first observation is that the effect of opening the windows is significant to reduce the concentration. The concentration of nanorange aerosol in Fig. 5(a) is reduced by half with windows open, and the microsize aerosol has the same trend, as shown in Fig. 5(b). Also, it is remarkable to see that the aerosol response time is greatly shortened with windows open for aerosols of both size ranges. The reduced response time could be due to the promoted air mixing with windows open.

**FIG. 6.**
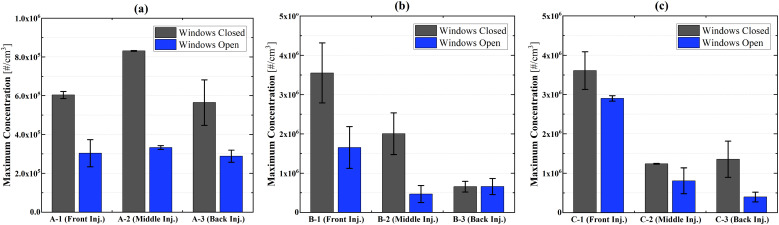
Nano-sized aerosol maximum concentration comparisons under window open and closed conditions: sampling at (a) the driver seat, (b) front seat, and (c) middle seat.

Figure 6 depicts the summary of all nine cases of experiments. Similar to case A-3 in Fig. 5, the maximum concentration is reduced by ∼50% when the windows are opened for all cases.

**FIG. 7.**
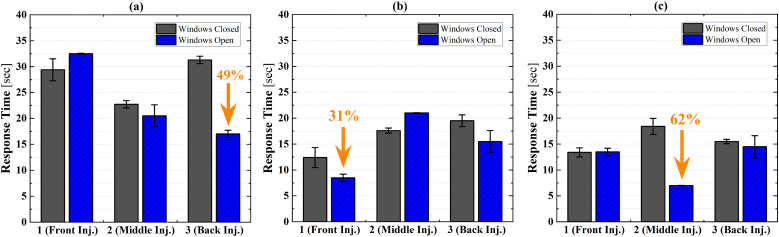
Response time comparison between window closed and open conditions: sampling at (a) the driver seat, (b) front seat, and (c) middle seat.

Figure 7 shows the summary of the response time with the windows open and closed. Although there are no significant variations between the window open and closed conditions, when the distance between the injection sampling locations is short, the response time is reduced with windows open.

## IV. COMPUTATIONAL ANALYSIS

The Reynolds-Averaged Navier–Stokes (RANS) equations are numerically solved to predict the turbulent flow field inside and around the bus. An energy equation is used to account for the influence of temperature variations. The virus-laden aerosols are modeled as a continuum in which the concentration density evolves according to its transport equation. The RANS equations together with the energy and aerosol concentration equations are solved using a customized solver based on the OpenFOAM open source CFD library.

### A. Numerical solver and governing equations

#### 1. Modeling of the airflow

The flow inside and around the bus is assumed to be incompressible and turbulent. The unsteady RANS equations represent the conservation of mass and momentum and are expressed as

∇⋅u=0, (2)

$$\frac{\partial u}{\partial t}+\nabla \cdot \left(uu\right)=-\nabla p_{rgh}-g\cdot x\nabla \left(\frac{\rho }{\rho_{0}}\right)+\nabla \cdot \left[\nu_{e\mathrm{ff}}\left(\nabla u+\nabla u^{T}\right)\right],$$ (3)

where **u** and *p*_rgh_ are the Reynolds-averaged velocity vector and kinematic pressure, **g** is the acceleration due to gravity, and *ν*_eff_ is the effective viscosity that accounts for both molecular and turbulent diffusion. The Boussinesq approximation is used in this model such that the density difference is ignored except in the gravitational term.^22^ The nominal air density is *ρ*_0_, and the local density is *ρ*.

The kinematic pressure represents the difference between the total pressure and the hydrostatic pressure, i.e., *p*_rgh_ = (*p* − *ρ***g** · **x**)/*ρ*_0_. The local density is determined from the local temperature according to *ρ*/*ρ*_0_ = 1 − *β*(*T* − *T*_0_). Here, *β* is the thermal expansion coefficient and takes a value of *β* = 3 × 10^−3^ K^−1^, and *T* and *T*_0_ are the local and nominal temperatures, respectively.

The temperature variations between the outside air, the cooled air coming from the air-conditioning system, and the passengers can generate flows due to buoyancy effects. As the temperature varies, so does the air density. For small variations in temperature (∼10 °C) and flow speeds much less than the speed of sound, the density variation can be neglected in the continuity equation and momentum equation with the exception of the gravity term (refer to pp. 117–121 in Ref. 23).

The equation governing temperature is

$$\frac{\partial T}{\partial t}+\nabla \cdot \left(uT\right)-\nabla \cdot \left(\alpha_{e\mathrm{ff}}\nabla T\right)=0,$$ (4)

where *α*_eff_ = *ν*_*t*_/Pr_*t*_ + *ν*/Pr and Pr_*t*_ = 0.9 and Pr = 0.71 are the turbulent and laminar Prandtl numbers, respectively. The *k* −*ε* turbulence model is used to determine the turbulent viscosity.^24^

#### 2. Modeling of aerosol transport

When humans breathe, cough, sneeze, sing, etc., small droplets are exhaled into the surrounding air. Under normal conditions such as breathing, speaking, and even coughing, most of the exhaled droplets are under 1 *µ*m in diameter and rarely larger than 5 *µ*m.^25,26^ This has significant relevance on the distance that an exhaled particle can travel. While larger particles are dominated by gravity and are pulled vertically downwards, the smallest particles are neutrally buoyant and move passively with the carrier fluid. To evaluate the role of gravity on the particle trajectory, the Stokes number of the droplets can be calculated as St = *τ*_*p*_/*τ*_*f*_, where the droplet kinematic timescale $\tau_{p}=\rho_{p}d_{p}^{2}/\left(18\nu \rho_{f}\right)$, with *ρ*_*p*_ and *ρ*_*f*_ being the densities of the particle and the fluid, respectively. For the majority of the exhaled droplets (*d*_*p*_ < 1 *µ*m), the droplet kinematic timescale *τ*_*p*_ < 1 *µ*s. In an indoor environment, the fluid timescale *τ*_*f*_ is larger than 1 s. The present numerical simulations focus on the transport of the droplets that travel passively with the carrier fluid, which are those with a diameter less than 5 *µ*m.

The transport of respiratory aerosols is complex and depends on different physical processes,^27^ including particle collisions, convection, diffusion, gravity, deposition, and evaporation. For example, the flow and aerosol transport in the lung are dominated by convection, diffusion, and deposition. The transport in a breathing alveolus of particles of the size 0.01 < *d*_*p*_ < 1 *µ*m is modeled with an Eulerian–Eulerian approach.^28^ The diffusion of submicron particles is modeled with a Stokes–Einstein coefficient that depends on the aerosol diameter. Aerosol transport in the lung can also be treated with particle-tracking methods. The study of convection, diffusion, and sedimentation of aerosols in a multigenerational acinar network of a lung^29^ shows that convection dominates for micrometer-size particles, and diffusion becomes important for submicron particles.

The transport and viability of aerosols just after exhalation depend on convection, diffusion, and evaporation. The ambient weather conditions such as temperature and humidity play a critical role in the viability of the virus after exhalation.^30^ A Eulerian–Lagrangian approach is used to study the influence of environmental conditions, and it is shown that the wind speed and high humidity contribute to the distance that the aerosols travel and significantly influence viability of the virus.^30^

The focus of the current work is the transport of micrometer-sized aerosols through a mechanically ventilated bus environment. The aerosols are considered as a passive scalar, and their transport is modeled with a convection–diffusion equation. The exhaled particles are described as an aerosol concentration of droplets per unit volume, *C*(**x**, *t*). The concentration field *C* is governed by the convection–diffusion equation,

$$\frac{\partial C}{\partial t}+\nabla \cdot \left(uC\right)-\nabla \cdot \left(D_{e\mathrm{ff}}\nabla C\right)=0,$$ (5)

where *D*_eff_ = *ν*_*t*_/Sc_*t*_ + *ν*/Sc and Sc_*t*_ = Sc = 1 are the turbulent and laminar Schmidt numbers, respectively. A Schmidt number of unity assumes that the aerosol diffuses at the same rate as momentum, which is relevant to 1 *µ*m–10 *µ*m particles under consideration, and thus, Brownian motion and other thermal effects on diffusion are neglected. This assumption is also used in the design of clean rooms for the manufacture of semiconductors.^31^

The equations governing the fluid flow, temperature, and aerosol concentration are solved using the OpenFOAM open source CFD library. A new solver is created based on buoyantBoussinesqPimpleFoam from OpenFOAM version 1906. The new solver includes an additional transport equation for the aerosol concentration. All discretization schemes are nominally second-order in space and time. The convection term in each transport equation is discretized with the second-order upwind scheme.

### B. Computational setup and case designs

The bus geometry, including the interior of the cabin, windows, doors, seats, hand rails, ventilation supply, and return (see Fig. 1), are determined from a laser scanner and used for generating the computational grid of the fluid domain. Manikins are placed at different locations inside the bus: a driver sitting behind the wheel and standing passengers. All simulations are conducted with a total of three people on the bus.

The infected passenger has a shedding rate of 50 s^−1^. A continuous breathing assumption is used so that the velocity on the mouth of the infected passenger is always outward at a breathing rate of 0.1 l/s. The continuous breathing model^10,32–34^ is appropriate for the analysis of the virus transport throughout the bus over the course of minutes. The mouth is modeled as a circle of diameter 0.04 m. For more detailed analysis of the flow near the mouth and the unsteady effects of inhalation and exhalation, an unsteady breathing model and particle tracking are required.^35^

A turbulence intensity of 2.5% and a turbulence length scale of 5 × 10^−3^ m are enforced at the supply vents for cooled air at 20 °C. For the breath of the passengers, the turbulence intensity and turbulence length scale are 10% and 7.5 × 10^−3^ m. A normal oral temperature of 37 °C is applied for the mouths of passengers as boundary conditions. The remaining surfaces of the cabin are assumed to be no-slip and adiabatic walls. Details of the boundary condition setup and relevant material parameters are summarized in Table III.

**TABLE III.** Boundary conditions and material properties for the simulation.

Boundary name	Boundary conditions
Passenger mouth	Velocity inlet/outlet, 0.1 m/s, 37 °C, 2.5% turbulence intensity, turbulence length scale is 5 × 10^−3^ m and *λ* = 50 s^−1^ for the infected passenger
HVAC supply vents	Velocity inlet, maximum rate is 5.4 m/s, 20 °C, 10% turbulence intensity and turbulence length scale is 7.5 × 10^−3^ m, circulate 80% of aerosols exiting through HVAC return
HVAC return	Pressure outlet
Seats, rails and cabin surfaces	No-slip and adiabatic walls
Material parameters	*ν* = 1.5 × 10^−5^ m^2^/s, *T*_0_ = 20 °C, *β* = 3 × 10^−3^ K^−1^, Pr = 0.71, Pr_*t*_ = 0.9, *g* = 9.81 m/s^2^, Sc = 1, Sc_*t*_ = 1

A precursor simulation of 3 min duration is performed to generate a fully developed turbulent flow field inside the passenger cabin. The resulting flow field is used for the initial condition for the simulation and analysis of the aerosol transport.

A series of simulations are performed to assess the numerical uncertainty, the influence of the HVAC system, the influence of the location of the infected passenger, and the roles of opening the windows and doors. The basic setup of all simulation cases is summarized in Table IV. In runs 1–8, windows and doors are kept closed, where different mesh resolutions and HVAC rates are applied to investigate the numerical uncertainty and the role of ventilation rates. The influence of the infected passenger’s location is considered by placing the passenger standing in the front or standing in the middle of the bus. In runs 9–12, all windows are kept open and the bus runs at a constant speed of 25 mph (40.23 km/h). Run 13 is designed to examine the effects of opening doors at bus stops.

**TABLE IV.** Case setup including the location of the infected passenger, number and location of susceptible passengers, grid resolution, and the ventilation rate represented by the percentage of the maximum HVAC flow rate.

Run No.	Infected passenger	Susceptible passengers	Grid	HVAC rate
1	Standing front	One standing rear	Coarse	Maximum
2	…	…	Medium	…
3	…	…	Fine	
4	…	…	Medium	50%
5	…	…	…	10%
6	Standing middle	…	…	Maximum
7	…	…	…	50%
8	…	…	…	10%
9	Standing front	…	Coarse	Maximum
10	…	…	Medium	…
11	…	…	Fine	…
12	Standing middle	…	Medium	…
13	Standing front	…	…	…

## V. RESULTS AND DISCUSSION

To visualize the flow field and spatial distribution of aerosol concentration, the results are displayed on two selected planes within the bus: a vertical plane at the centerline of the bus and a horizontal plane at the elevation of the mouths of the passengers standing on the front platform or sitting on rear seats.

The number of inhaled particles is adopted as a cumulative point of view to define the risk of each person as^9^

$$N_{b}\left(t\right)=\int_{0}^{t}C{\dot{V}}_{b}\;d\tau ,$$ (6)

where ${\dot{V}}_{b}$ is the human breathing rate, and we assume the average rate as ${\dot{V}}_{b}=0.33\;{dm}^{3}s^{-1}$. While it is still unclear how much dose of virus is needed for someone to be infected,^36^ we use a conservative assumption of *N*_b,crit_ = 50. This value is based on the work of Kolinski and Schneider^37^ where they analyzed 20 reported superspreading events during the ongoing pandemic.

Heat transfer and evaporation modeling are a critical part of predicting virus transmission. Zang *et al.*^38^ reviewed and studied various applications of droplet evaporation including droplets containing biological matter, and they emphasized that the complexity in this problem arises from the multi-scale nature of the process. Dbouk and Drikakis^35^ show that high temperature and low relative humidity lead to high evaporation rates of virus-laden aerosols, which reduces the virus viability. The present analysis neglects the effects^39–46^ of heat transfer and evaporation modeling and thus represents a conservative analysis of the risk of transmission.

### A. Refinement study

The computational mesh is comprised of finite volumes that are dominantly hexahedral. Local refinement is used around fine features, namely, the HVAC supply vents, the manikins, and parts of the bus surface such as seats, windows, and handrails.

**FIG. 8.**
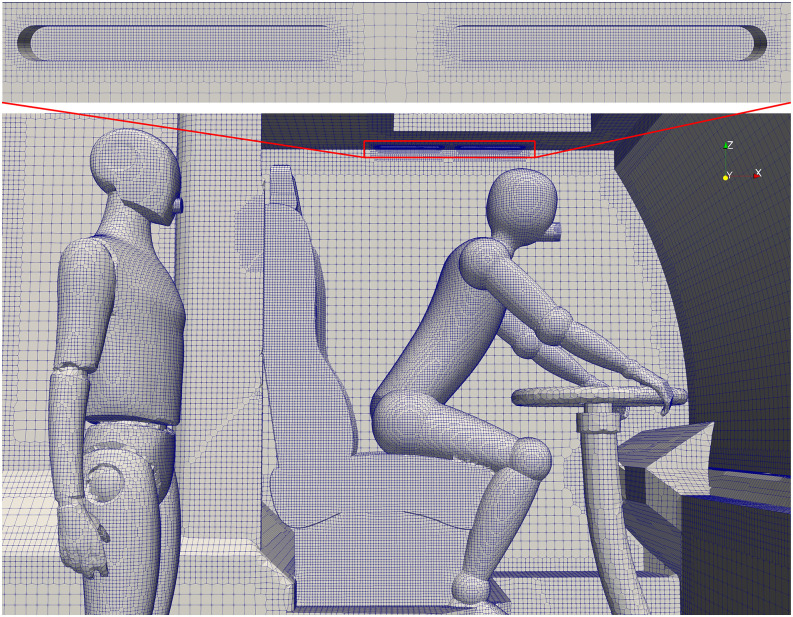
Numerical grid in front of the cabin with the close-up of the HVAC supply vent.

A grid refinement study is performed to assess the sensitivity of the results on the numerical discretization. Three grids with resolutions of 250 mm, 125 mm, and 62.5 mm in the bulk of the flow domain are adopted as the coarse, medium, and fine grids, with a total number of cells of 2.04, 5.87, and 11.65 × 10^6^, respectively. The supply vents and mouths of people have the smallest cell sizes of 2 mm and 4 mm, which are the same for all three grids. The time step size is set based on a maximum Courant number of 25, which corresponds to the step size of ∼0.005 s for each simulation at the maximum ventilation rate. An image of the medium grid is shown in Fig. 8.

**FIG. 9.**
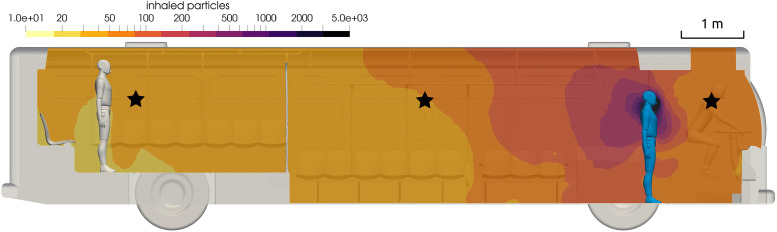
Contour of inhaled particles on the center plane of the bus, *t* = 15 min. Black stars indicate the probe locations.

The number of inhaled particles after 15 min for each location on the bus for fine grid run 3 is shown in Fig. 9. Note that the contours are spaced with logarithmic scaling. The infected passenger is placed at the front of the bus. The darker colors near the front passenger indicate that if another passenger was positioned there, the number of particles inhaled in 15 min would be much greater than the assumed threshold of 50. In the back of the bus, the number of inhaled particles is less. The primary mechanisms that set the distribution of particles through the bus are convection due to the air currents of the HVAC system, the mixing due to turbulence, and the dilution due to the addition of fresh air in the HVAC system.

**FIG. 10.**
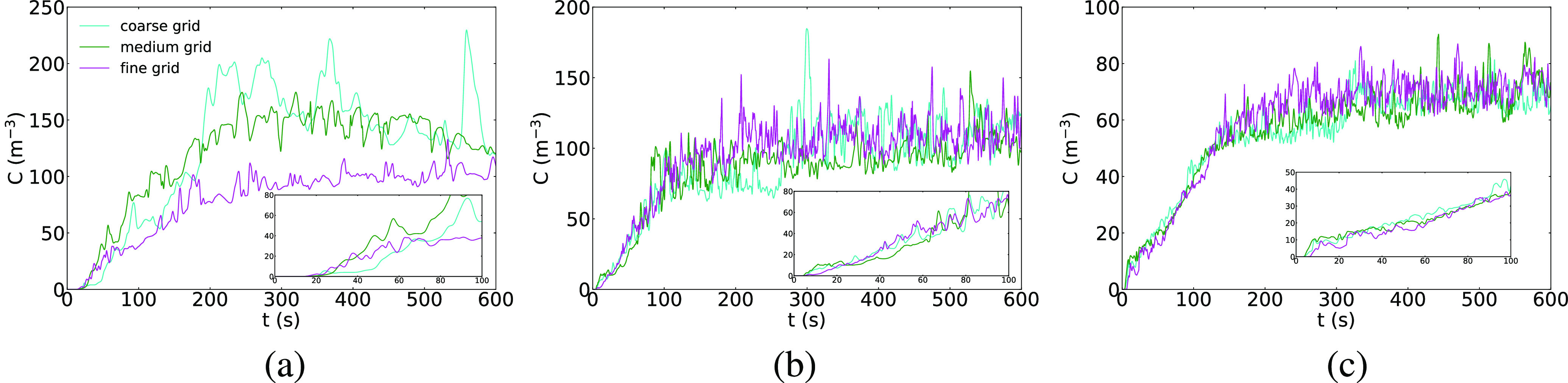
Time histories of concentration with different grid resolutions. (a) Front probe, (b) middle probe, and (c) rear probe.

The time histories of concentration at three locations in the bus are shown in Fig. 10. The locations are shown as black stars in Fig. 9. Inspection of the time histories shows that the three grids predict the same concentration field at all three locations, although the small differences are greatest for the probe in the front of the bus. Also, the time history shows that the equilibrium concentration is reached after ∼150 s for the middle probe and after 200 s for the rear probe. This indicates that even a short trip on a bus can present exposure to a passenger, although the quantity of inhaled particles will be small at first and grow with time. The results in Fig. 10 can be compared to the experimental measurements in Fig. 7. In the experiment, the response time is calculated as the time between the start of smoke generation and the initial rise in concentration, and under conditions with the windows closed, this quantity is between 10 s and 30 s. The grid refinement data are replotted over the range of 100 s to show that the response time is similar. It is interesting to note the largest rise time is for the position in the front of the bus. This is due to the lower convection velocity from the HVAC, and both the experiment and simulation are in agreement with this point. Based on the analysis of runs 1–3, the medium grid is used for the rest of the runs for the window-closed simulations.

### B. Flow-field inside bus

**FIG. 11.**
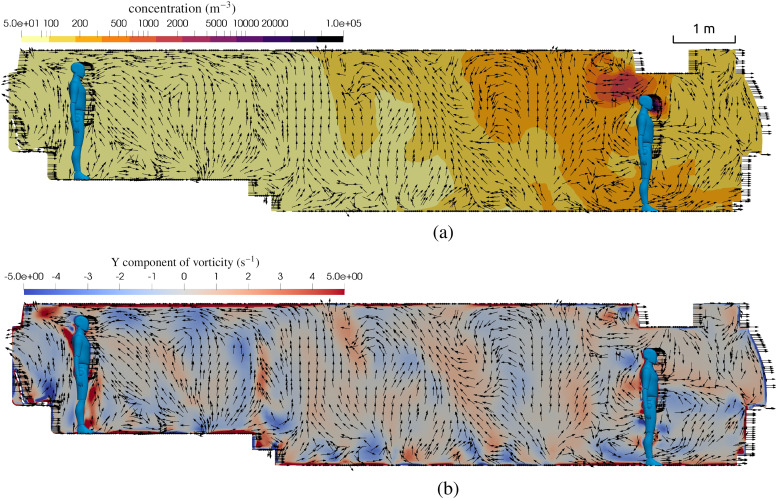
Flow field details on the center plane of the bus with windows closed and HVAC at the maximum rate. (a) Contour of aerosol concentration and velocity vectors, *t* = 15 min. (b) Contour of vorticity and velocity vectors, *t* = 15 min.

Turbulence is a primary transport mechanism for aerosols, and it depends on the geometry of the passenger compartment, the opening of doors and windows, and the HVAC system. The high-resolution simulations used in this work allow for inspection of the dominant flow features in the passenger compartment. Figure 11 shows the velocity vector field on the center plane together with the concentration field (top) and the vorticity field (bottom). It can be seen that the turbulent flow moves both up and down as the net flow is rearward through the compartment. The highest values of vorticity are observed near corners and the supply vents, which aid in mixing aerosol concentration.

### C. Risk under different ventilation rates

The HVAC system is a primary aspect of the transport of aerosols within the bus (this is true for many confined spaces), and it is important to quantitatively assess how the exposure varies as the HVAC fan speed is changed. The HVAC system adds fresh air as a fraction of its flow rate (in this case 20%), and it acts to mix and transport the smallest particles through the cabin.

**FIG. 12.**
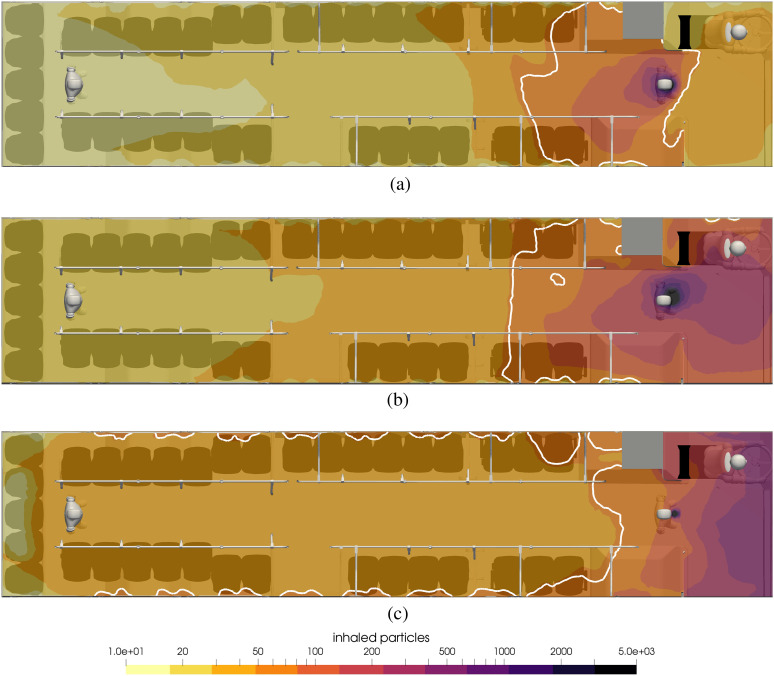
Contours of inhaled particles for different HVAC rates with the infected passenger standing in the front of the bus at t = 15 min. The white contour lines represent the critical number of inhaled particles *N*_b,crit_ = 50. (a) Maximum HVAC rate. (b) 50% of the maximum rate. (c) 10% of the maximum rate.

**FIG. 13.**
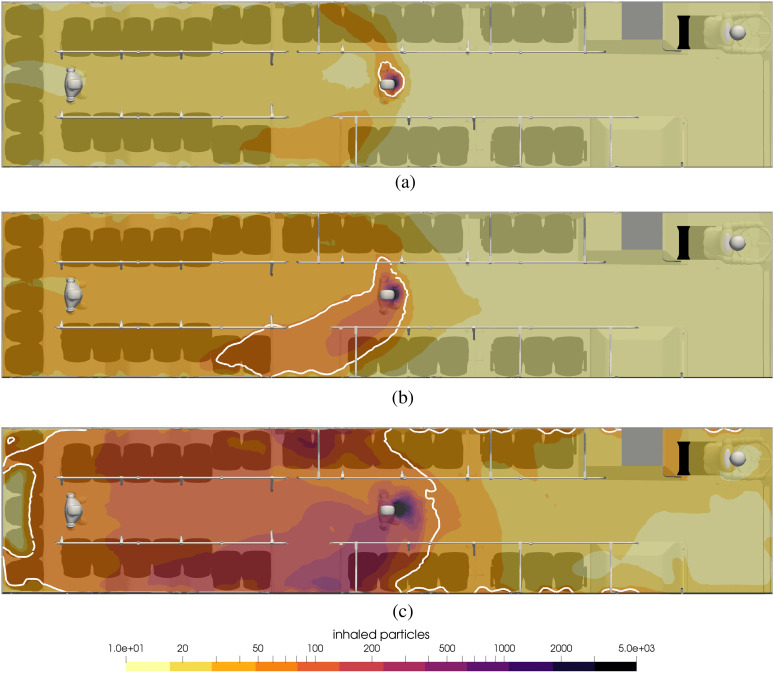
Contours of inhaled particles for different HVAC rates with the infected passenger standing in the middle of the bus at t = 15 min. The white contour lines represent the critical number of inhaled particles *N*_b,crit_ = 50. (a) Maximum HVAC rate. (b) 50% of the maximum rate. (c) 10% of the maximum rate.

Figures 12 and 13 shows the contours of inhaled particles for three different HVAC settings: the maximum flow rate, 50% of the maximum, and 10% of the maximum. In Fig. 12, the infected passenger is at the front of the bus, and in Fig. 13, the infected passenger is in the middle. The contour representing the inhalation of 50 particles is shown in the thick white line such that inside these contours, a passenger would inhale more than 50, and outside them, they would inhale less. Hence, a count of the number of seats or standing positions inside the area bounded by the white line indicates the number of transmissions in the 15 min exposure time.

In Fig. 12, it can be seen that as soon as the HVAC rate is reduced to 50%, the region of elevated risk grows substantially and covers the entire front of the bus. The case with the infected passenger in front poses serious risk to the driver, especially considering that the driver is on the bus for extended periods of time.

Figure 13 shows the influence of the HVAC flow rate for the infected passenger in the middle of the bus. A similar effect is seen where a reduction in the fan speed enhances the risk to surrounding passengers, and while the area of greater than 50 particles is relatively small for the 50% fan speed, for the lowest fan speed, nearly all passengers to the rear of the infected passenger could be infected during the 15 min trip. In this case, the driver is relatively safe since the single HVAC return vent draws air toward the rear of the bus and effectively isolates the driver. Note that for the lowest flow rate, while the transport of the aerosols is primarily rearward, there is transport forward of the infected passenger. This is due to the chaotic nature of the turbulent flow, as well as diffusion, which becomes more important as the ambient air currents lessen in intensity. Also, while the HVAC adds 20% fresh air, it does take the virus-laden air and returns it throughout the HVAC supply vents that are located from the front to rear (this is seen in the white contours near the supply vents).

To summarize the numbers of transmissions in the 15-min exposure, when the infected passenger is at the front of the bus, there will be 3, 5, and 2 transmissions for the 100%, 50%, and 10% HVAC rates, respectively. The numbers are 0, 3, and 26 when the infected passenger stands in the middle of the bus.

### D. Effects of face masks

Face masks (or face coverings) are a primary line of defense for reducing COVID-19 transmission. Many researchers around the world are working to scientifically quantify the effects of wearing masks. In this work, we use a simple model for a mask based on the work of Ref. 19 in which the fraction of exhaled particles is predicted using CFD. Face masks are found in different types, and in this work, two masks, a surgical mask and a handmade mask, are analyzed. We assume that the surgical mask will block 90% of the exhaled and inhaled aerosols, and the handmade masks block 30% of the particles.

**FIG. 14.**
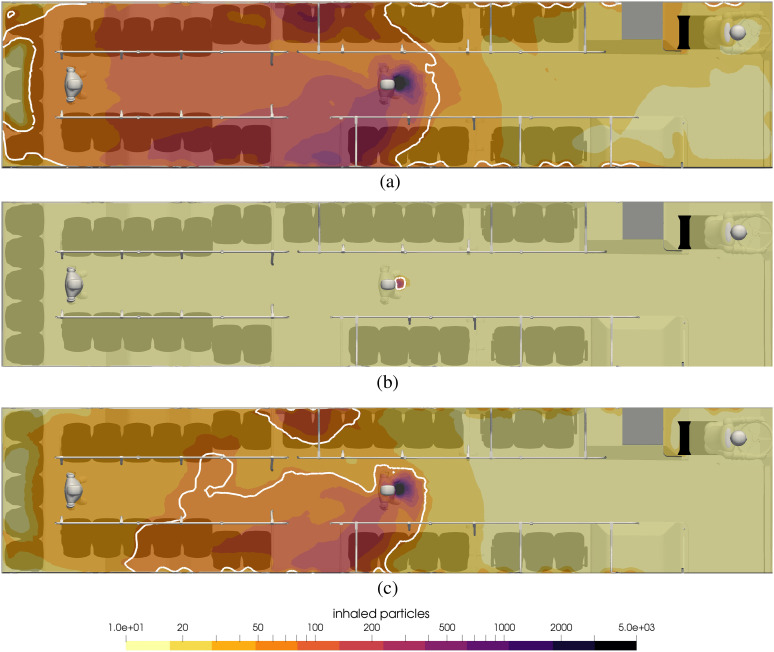
Contours of inhaled particles for different scenarios of face coverings at t = 15 min. The white contour lines represent the critical number of inhaled particles *N*_b,crit_ = 50. (a) Nobody wears a mask, (b) everyone wears a surgical mask, and (c) everyone wears a handmade mask.

Figure 14 shows the contours of inhaled particles for the cases of no mask (top), everyone with a surgical mask (middle), and everyone with a handmade mask (bottom). It is impressive to see how the surgical mask significantly reduces the number of inhaled particles. In the top figure with no mask, nearly all passengers to the rear of the infected passenger inhale more than 50. On the other hand, when everyone wears a surgical mask, during the 15 min ride, not a single passenger inhales anywhere near 50 particles, and unless the susceptible person is standing face-to-face with the infected person, the number of inhaled particles is less than 2.

In the bottom of Fig. 14, the number of particles for a handmade mask is shown. Clearly, the effect of wearing a mask is to reduce the number of aerosols that are inhaled, although in this case, there are still several people that could be inside the white contour. Also, for passengers throughout the rear portion of the bus, the effect of handmade masks is to reduce the number of particles from more than 50 in the case without masks to a number as low as 20. For this case, 26 seated passengers will be infected during the 15-min ride if no one wears a mask, and the number will be 0 if both the infected and susceptible passengers wear surgical masks and 10 if both wear handmade masks.

### E. Effects of opening windows and doors

An important mechanism for reducing the aerosol concentration is to add fresh air. This can be done manually by adjusting the HVAC system or passively when the doors and windows are open on the bus. To quantify the effect of opening windows and doors, simulations are conducted with the bus moving at 25 mph (40.23 km/h) with the windows open. Also, a simulation is done with the windows closed but with the doors open for 30 s at five stops during the 15 min trip. For the case with doors open, there is a 5 mph (8.05 km/h) wind blowing opposite to the direction of travel of the bus.

**FIG. 15.**
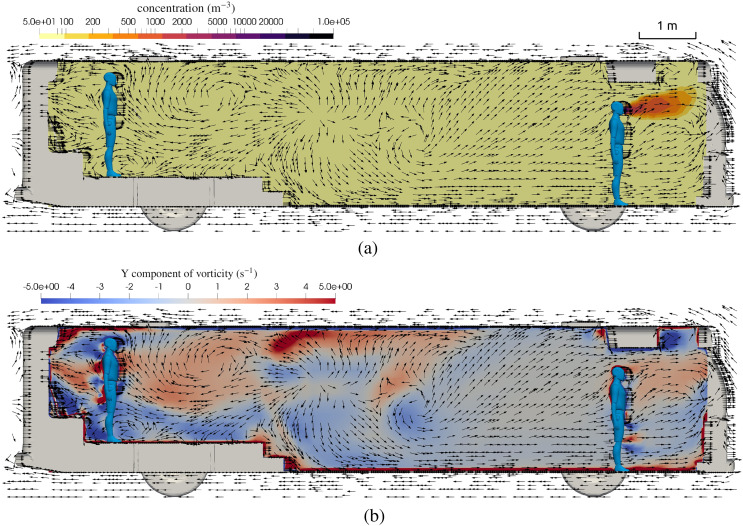
Flow field details on the center plane of the bus with windows open. (a) Contour of aerosol concentration and velocity vectors, *t* = 15 min. (b) Contour of vorticity and velocity vectors, *t* = 15 min.

The primary difference when the doors and windows are open is that fresh air can be added (or virus-laden air removed), and the flow field can be materially different. Figure 15 shows the flow field inside the bus with the windows open. This figure should be compared to Fig. 11 where the windows are closed. The most notable difference is that when the windows are open, there is a net rearward flow for the middle of the bus and behind, but there is a net outflow through the driver window, which draws air forward. This highlights the complicated nature of turbulent flows within occupied spaces and how small changes can significantly alter the flow and hence the risk of transmission. While in the aggregate, the risk is reduced for passengers when the windows are opened, the risk to the driver has been increased when the infected passenger is standing up front in the bus. Similar to the window closed flow field, the movement of aerosols has both upward and downward motion that mixes and renders risk the same whether one is seated or standing.

**FIG. 16.**
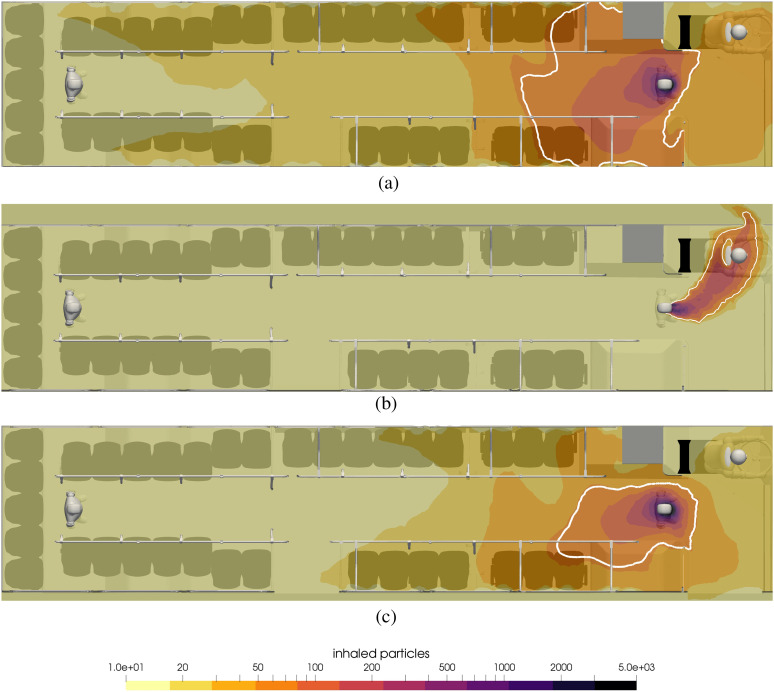
Contours of inhaled particles with different setup for the windows and doors at t = 15 min. The white contour lines represent the critical number of inhaled particles *N*_b,crit_ = 50. (a) Bus is enclosed, (b) windows are open, and (c) doors are open at each stop.

Figure 16 summarizes the results for the effects of opening windows and doors. At the top of this figure, the result for a single passenger without the facemask is shown, and in the middle, the contour of inhaled particles for the windows open and, in the bottom, for the case when the windows are closed, yet the doors open periodically. Here, it is clearly seen how, overall, the number of inhaled particles decreases significantly throughout the passenger compartment, with the exception of focusing of virus-laden air in front of the driver.

The influence of opening the doors is seen to slightly reduce the number of particles inhaled throughout the bus.

To summarize the numbers of transmissions in the 15-min exposure, there will be three transmissions in the enclosed cabin: one transmission (the driver) if windows are open and one transmission if doors open periodically.

## VI. CONCLUSIONS

In this paper, a detailed analysis of the airborne transmission of respiratory aerosols is conducted using the experimental measurement and computational fluid dynamics. The transmission on an urban bus is studied to identify the transmission mechanisms and to assess strategies to reduce risk. Specifically, risk is quantified and the effects of the air-conditioning system, opening windows and doors, and wearing masks are analyzed.

Experiments are performed on a campus bus of the University of Michigan. The temporal history of concentration and size of particles emitted from a smoke generator are measured for a variety of particle injection and sampling locations throughout the bus. The effects of opening doors and windows are quantified.

Numerical simulations are performed with a highly infectious passenger aboard the bus, and the exhaled aerosols are modeled as a concentration field. The transport of the aerosol concentration is determined by the solution of the turbulent flow within the passenger compartment and a transport equation for the concentration. A risk metric of the number of particles inhaled by susceptible passengers is defined so that different risk mitigation strategies can be compared and assessed quantitatively.

The analysis shows that under the condition of the HVAC system at its maximum setting, the airflow in the bus is turbulent and the time scales of transit from an infected passenger to any susceptible passenger are less than a minute. The HVAC flow rate and bus interior volume correspond to ∼60 air-changes/h. While such a high air-exchange rate is desired, it also means that six-foot spacing does not protect a susceptible passenger. While the short response time appears to increase risk, the HVAC actually reduces risk because the turbulence mixes the aerosols with the ambient air, thereby reducing concentration, and the HVAC system adds fresh air that, thus, dilutes the concentration further.

The effect of opening doors and windows is to reduce the concentration by approximately one half. The CFD analysis shows that for almost all passengers, this is true, while care should be exercised that in certain cases, the outflow of contaminated cabin air could pass by a passenger (in this case, it is the driver) and increase the risk to those near the outflow window or door.

A mask model is used to quantify and visualize their influence. It is shown that well fitted surgical masks, when worn by both infected and susceptible passengers, can nearly eliminate the transmission of the disease. In the case of poorer quality masks, their effect is still to reduce transmission for all aboard the bus.

## DATA AVAILABILITY

The data that support the findings of this study are available from the corresponding author upon reasonable request.
